# Traitement conservateur des omphalocèles géantes par l’éosine aqueuse disodique 2%: une série des cas

**DOI:** 10.11604/pamj.2021.39.63.23215

**Published:** 2021-05-21

**Authors:** Trésor Kibangula Kasanga, Tshiband Mosh Bilond, Florent Tshibwid A Zeng, Hugor Mujinga Wa Mujinga, Augustin Kibonge Mukakala, Nathalie Dinganga Kapessa, Éric Mbuya Musapudi, François Katshitsthi Mwamba, Prince Muteba Katambwa, Dimitri Kanyanda Nafatalewa, Israël Tshiamala Badypwyla, Stephanne Ilunga Mukangala, Christelle Ngoie Ngoie, Vincent De Paul Kaoma Cabala, Manix Ilunga Banza, Sébastien Mbuyi Musanzayi

**Affiliations:** 1 Département de Chirurgie, Faculté de Médecine, Cliniques Universitaires de Lubumbashi, Université de Lubumbashi, Province du Haut-Katanga, République Démocratique du Congo

**Keywords:** Omphalocèle géante, traitement conservateur, éosine aqueuse, Giant omphalocele, conservative treatment, aqueous eosin

## Abstract

La fermeture chirurgicale primaire dans le traitement de l´omphalocèle géante est émaillée des complications. Le traitement conservateur est une option adaptée aux pays à faible revenu où la chirurgie te la réanimation néonatales sont pourvoyeuses d´une grande mortalité. Ceci est une étude prospective menée aux cliniques universitaires de Lubumbashi, incluant les patients reçus entre janvier et avril 2020 et qui ont bénéficié d´un traitement conservateur à l´éosine aqueuse disodique selon un protocole défini. Trois patientes ont été inclues dans notre série. L´âge moyen était de 24 heures (1 - 48), toutes nées à terme (38 - 39 SA), et par voie basse, sans aucun diagnostic anténatal posé. La moyenne du poids de naissance était de 2.800 grammes (2.400 - 3.000). Le diamètre moyen du sac était de 13,7 cm (11 - 15 cm), le sac contenant le foie dans tous les cas. Le délai moyen de nutrition entérale était de 4,3 jours (4 - 5 jours), celui de granulation était de 31,7 jours (30 - 33 jours) et celui d´épithélialisation était de 71,7 jours (60 - 90 jours). Aucun décès n´a été déploré. Ces résultats préliminaires encouragent l´utilisation de l´éosine aqueuse disodique dans le traitement conservateur des omphalocèles géantes non rompues.

## Abstract (en)

Primary surgical closure for the treatment of giant omphalocele is punctuated by the onset of unpleasant complications. Conservative treatment is an option in low-income countries where neonatal resuscitation is associated with high mortality rates. We conducted a prospective study of patients admitted to the University Clinics of Lubumbashi between January and April 2020 and receiving conservative treatment based on dissodic 2% aqueous eosin according to a defined protocol. Three patients were included in our series. The mean age was 24 hours (1 - 48); they were all full term newborns (38 - 39 SA), born vaginally and with no prenatal diagnosis. Mean birth weight was 2,800 grams (2,400 - 3,000). Mean amniotic sac diameter was 13.7 cm (11 - 15 cm); it contained the liver in all cases. The median time to enteral feed was 4.3 days (4 - 5 days), to granulation was 31.7 days (30 - 33 days) and to epithelialization was 71.7 days (60 - 90 days). No death was reported. These preliminary results encourage the use of disodium aqueous eosin for the conservative treatment of unbroken giant omphaloceles.

## Introduction

L´omphalocèle est une anomalie congénitale de la paroi de l´abdomen, siégeant au niveau de la région ombilicale résultant de la non-réintégration de l´anse primitive dans la cavité abdominale après la hernie physiologique autour de la 5^e^semaine. Les omphalocèles peuvent être classées en géantes (larges ou majeures) et en mineures. Bien qu´il n´existe pas de consensus sur la définition des omphalocèles géantes (OG), les différents critères, selon les diverses définitions, incluent souvent: une omphalocèle trop large pour être traitée par fermeture primaire, celle dont le collet est > 5 cm ou celle dont le sac contient le foie [1, 2].

Si le traitement des omphalocèles mineures peut se faire par la fermeture primaire, cela n´est généralement pas envisageable pour les OG, au risque d´induire un syndrome de compartiment intra-abdominal, lequel entamerait le pronostic vital [3]. Plusieurs moyens de traitement ont été proposés, parmi lesquels, les moyens chirurgicaux et ceux conservateurs. Les moyens chirurgicaux nécessitent, soit un monitoring respiratoire postopératoire (technique de Gross), soit un silo et une alimentation parentérale (technique de Schuster), lesquels sont rares et couteux pour les pays à faibles revenus [3]. Le traitement conservateur vise la formation d´une escarre, suivie de son épithélialisation, transformant ainsi l´OG en une éventration. Ce traitement est associé à de bons résultats : meilleure survie, réduction de la durée d´hospitalisation, réduction du délai de l´alimentation entérale complète et suppression des complications associées à la fermeture précoce [4]. Pour ce fait, plusieurs substances ont été appliquées sur le sac de l´OG, avec chacune, des avantages et inconvénients dont le plus à craindre est une intoxication à la substance de tannage. Nous présentons ici une série préliminaire de 3 patientes ayant bénéficiés d´un traitement conservateur à base d´éosine aqueuse disodique (EAD) 2%.

## Méthodes

### Type d´étude et cadre

Il s´agit d´une étude descriptive transversale prospective. Elle s´est déroulée de janvier à avril 2020, dans le service de chirurgie des Cliniques Universitaires de Lubumbashi.

### Critères d´inclusion et d´exclusion

Nous avons inclus les patients diagnostiqués d´omphalocèle géante reçu dans notre service au cours de notre période d´étude. Ont été exclus tous les patients avec omphalocèle mineure, omphalocèle majeure traitée par une autre méthode, et les omphalocèles géantes rompues. A cet effet, trois patientes ont été retenues dans notre étude.

### Récolte et analyse des données

Les données ont été enregistrées sur une fiche d´enquête et encodées sur un tableau Excel et analysées ave EPI Info. Les variables récoltées ont concerné les éléments sociodémographiques, les circonstances de l´accouchement, l´aspect de l´omphalocèle, le traitement et les résultats du traitement.

### Protocole de prise en charge

**Figure 1:**
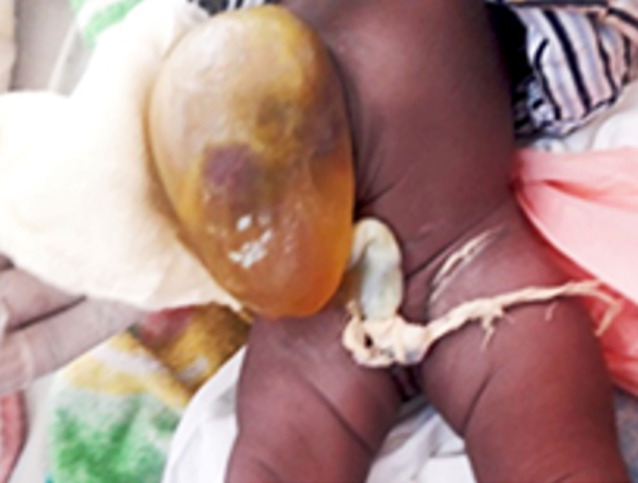
état initial

La prise en charge a consisté en: 1) une administration parentérale d´antibiotiques: Céfotaxime 100 mg/kg x 3/jr et Ampicilline 100 mg/kg x 3/jr. 2) Les besoins énergétiques et hydriques ont été comblés avec du SG 10%. 3) L´alimentation entérale a été initiée au quatrième jour, et poursuivie en absence de résidus dans la sonde nasogastrique. 4) Localement, nous avons appliqué l´éosine aqueuse selon le protocole suivant:

Initialement (Figure 1), nous avons effectué des pansements humides à l´éosine aqueuse 2%, 2 ml dilués dans 500 ml sérum salé isotonique à raison de 100 ml du mélange par pansement et ce, trois fois par jour.

**Figure 2:**
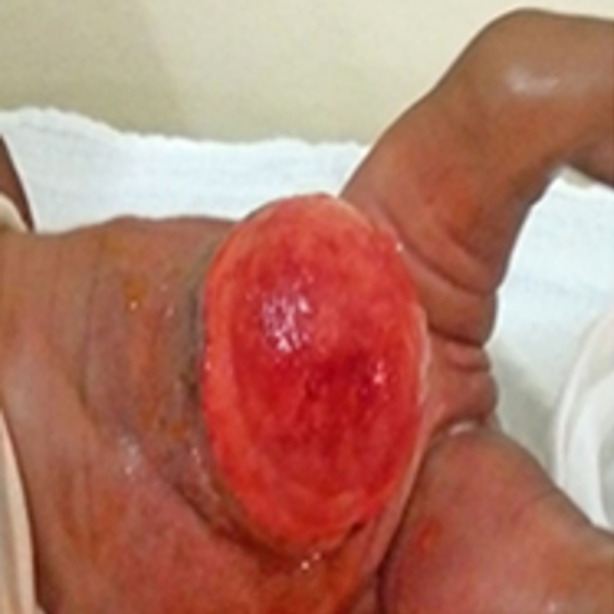
tissu de granulation

Dès l´obtention du tissu de granulation (Figure 2) témoignant de la fin de l´escarrification de la membrane de l´omphalocèle, nous avons réduit le nombre de pansement à l´éosine par jours, avec 100 ml du mélange. Jusqu´à l´obtention du tissu de granulation, le patient était hospitalisé.

**Figure 3:**
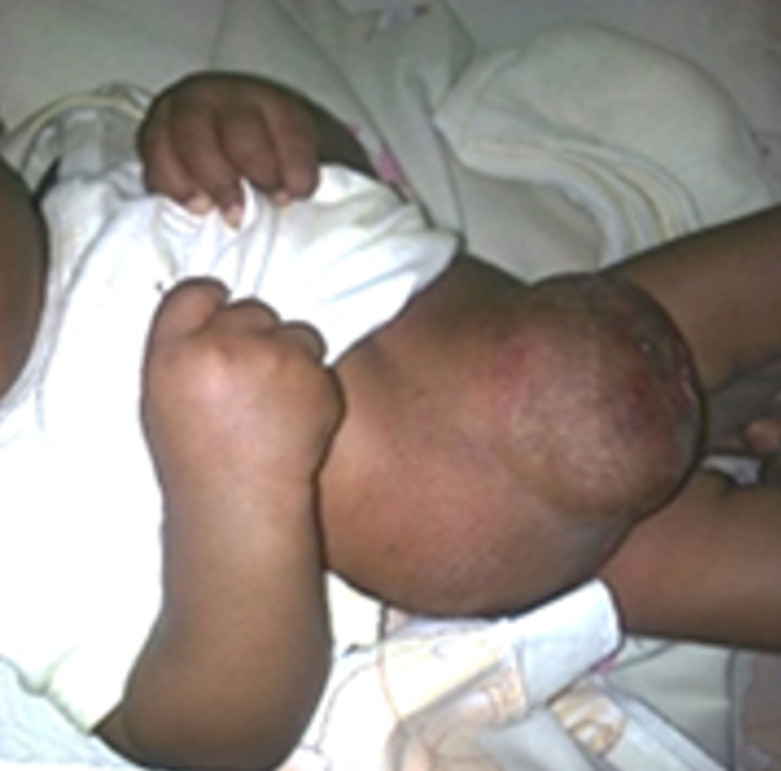
phase d´épithélialisation complète ou de cicatrisation complète

Dès le début de l´épithélialisation, nous avons fait le tannage à l´éosine aqueuse 2% seul, deux fois par jour et laissé à l´air libre. Cette étape se fait en ambulatoire en prenant la précaution d´asepsie, en fin phase de cicatrisation complète (Figure 3).

## Résultats

### Caractéristiques socio-démographiques

Les trois patientes (P1, P2 et P3) étaient respectivement âgées de 24 heures, une heure et 48 heures. Elles sont toutes nées par voie basse, à terme 38 semaines d´aménorrhée (SA) 4 jours pour P1, 38 SA pour P2 et 39 SA pour P3. Les trois accouchements ont été faits dans des centres médicaux de références, sans réanimation néonatale (score d´Apgar de 8/9/10, 8/9/10 et 9/10/10 respectivement). Les poids de naissance ont varié de 2400 grammes pour P1 à 3000 grammes pour P2 et P3. A noter qu´aucune patiente n´a bénéficié d´un diagnostic anténatal.

### Eléments cliniques

Le diamètre du collet a varié de 11 cm pour P1 et 15 cm pour P2 et P3. Le sac contenait des anses intestinales et le foie chez toutes les patientes, ainsi que d´autres viscères difficilement identifiables pour P1 et P2. Aucune malformation associée n´a été identifiée.

### Traitement

Toutes les patientes ont bénéficié d´un traitement médical et local selon le protocole présenté plus haut.

### Résultats du traitement

Le délai d´initiation de la nutrition parentérale a été de 4 jours pour P1 et P3, et 5 jours pour P2. Le délai d´escarrification, qui correspond à la durée d´hospitalisation, a été de 30 jours pour P1, 33 jours pour P2 et 32 jours pour P1. Le délai d´épithélialisation a été de 60 jours pour P1, 90 jours pour P2 et 65 jours pour P3.

## Discussion

Bien qu´il n´existe pas de consensus sur la définition de l´OG, plusieurs auteurs s´accordent sur le fait que toute omphalocèle qui ne peut être réduite par une fermeture primaire est une OG. De ce fait, le jugement clinique du chirurgien devient l´élément important, permettant d´évaluer la disproportion viscéroabdominale et de là, prédire l´impossibilité d´une fermeture primaire [1]. Pour notre part, nous avons considéré les éléments proposés par *Aitken J*. (Tableau 1) et considéré le type 2 comme étant la définition de l´OG [5]. De nos jours, la classification d´Aitken demeure la plus utilisée [6].

**Tableau 1:** classification des omphalocèles selon Aitken

Type	Critères
1 (tous les critères doivent être présents)	Diamètre du collet (défect du fascia) < 4 cm
	Diamètre du sac < 8 cm
	Absence du foie dans le sac
2 (un seul critère suffit)	Diamètre du collet (défect du fascia) > 4 cm
	Diamètre du sac > 8 cm
	Présence du foie dans le sac

La prise en charge des OG peut faire intervenir plusieurs moyens non conservateurs, avec le but d´obtenir une fermeture faciale le plus tôt possible. Il s´agit de la fermeture fasciale primaire (primary closure) et de la fermeture fasciale par étapes (staged closure) selon la technique de Gross ou de Schuster dont plusieurs modifications ont été proposées [7]. Ces méthodes chirurgicales sont difficilement réalisables dans notre milieu à cause de plusieurs raisons: le manque des moyens importants de surveillance pour guetter un syndrome de compartiment intra-abdominal et la prise en charge des patients nécessitant une ventilation mécanique, la rareté et le coût du matériel prothétique pour aboutir à une fermeture complète du défect fascial, du coût l´alimentation parentérale nécessaire avant la reprise de l´alimentation entérale complète, la multiplicité des interventions chirurgicales dans le but d´aboutir à une fermeture faciale totale [7, 8].

Le traitement conservateur (delayed closure) des OG consiste en l´application d´une substance escarrifiante sur la membrane de l´omphalocèle, avec comme conséquence l´escarrification, suivie de la granulation et enfin, de l´épithélialisation centripète à partir des bords cutanés. En dehors des propriétés escarrifiantes, la substance doit avoir des propriétés antimicrobiennes, qui permettent de lutter contre l´infection du sac [4]. Il en résulte une éventration de taille variable dont la réparation se fait entre 3 et 5 ans, selon les milieux. Il a été décrit la première fois en 1899 par Ahlfeld qui a utilisé des pansements à l´alcool et ce n´est qu´en 1957 que Grob a décrit l´utilisation d´une solution de 2% de merbromine (mercurochrome) [9]. Dès lors, plusieurs substances de tannage ont été proposées, certaines ont rapidement été abandonnées à cause de l´intoxication entrainée par leur absorption au niveau de la membrane du sac ayant abouti au décès dans certains cas. Le mercurochrome est le plus dangereux et le premier décès imputable à son usage a été rapporté dès les années 70 [10]. Le nitrate d´argent a aussi été abandonné, de même que l´alcool, à cause des cas d´intoxications rapportées [11].

La dysthyroïdie causée par l´absorption d´iode par application de providone iodée est encore largement discutée. Si des cas d´hypothyroïdie ont été rapportés, des études plus récentes n´ont pas rapporté cette complication et ce, même en effectuant des dosages réguliers [4, 9, 11, 12]. D´autres substances sont utilisables sans le moindre danger, il s´agit de la sulfadiazine argentique, le miel Manuka, le violet de gentiane et l´éosine aqueuse disodique 2%. Récemment, l´utilisation de la fermeture par pression négative a été décrite [7, 13-15]. Une récente méta-analyse a montré que, par rapport au traitement chirurgical, le traitement conservateur est associé à une faible mortalité et une plus courte durée d´alimentation parentérale, ce qui pourrait promouvoir un meilleur développement neurologique à long terme [15].

L´utilisation de l´éosine aqueuse comme escarrifiant a été rapportée par Kouame BD *et al*. dans une large série de 175 patients sur 15 ans. Les résultats, en termes de durée d´épithélialisation, de morbidité et de mortalité, se sont révélés semblables à ceux des études ayant utilisé d´autres topiques [14, 15]. En comparaison aux autres topiques, l´EAD a une plus faible durée d´hospitalisation car les parents peuvent continuer le reste des applications à domicile, dès le début de l´épithélialisation [15]. Par rapport au traitement chirurgical des OG, le traitement conservateur à l´EAD 2% est aussi associé à une faible durée de nutrition parentérale, à un court délai de tolérance de l´alimentation entérale, à une courte durée d´hospitalisation en soins intensifs et en unité de chirurgie [16].

Malgré l´évolution des moyens de prise en charge, tant au niveau de la réanimation néonatale, de la chirurgie, de l´anesthésie et de la nutrition, les OG restent un challenge pour le chirurgien. La mortalité est autours de 25% selon différentes séries et est fortement associée à la présence des anomalies chromosomiques et des malformations associées, lesquelles peuvent être rencontrées jusqu´à 50% des cas [7]. Il s´agit principalement des malformations cardiaques, pulmonaires et intestinales, et parfois regroupées en syndromes dont les plus décrits sont le syndrome de Beckwith-Wiedemann et la pentalogie de Cantrell. Les autres éléments impactant la morbidité et la mortalité sont la taille du défect, la rupture in utero du sac, le faible poids de naissance et la détresse respiratoire périnatale [4, 16]. En l´absence d´anomalies chromosomiques et des malformations associées sévères, la mortalité des OG est réduite [17]. L´absence des malformations congénitales associées a probablement contribué à l´absence de décès parmi nos patients.

La fermeture faciale avec réparation de l´éventration est généralement programmée à l´âge de 6 à 12 mois selon de nombreux auteurs [18]. Cependant, dans 25% des cas, il y a nécessité d´utiliser une prothèse pour aboutir à une fermeture complète du défect dans le fascia [8]. Ces dispositifs coûtent chers et ne sont pas couverts par une assurance santé dans les pays en développement. Des auteurs ont proposé l´utilisation du sac de l´éventration comme matériel de substitution pour complètement fermer le défect à la place d´un matériel purement synthétique. Dans ce cas, la réparation est programmée dans 2 ans, le temps de s´assurer que le sac est bien rigide [19]. Pour notre part, nous avons fixé l´âge à 5 ans en comptant sur et donc, une fermeture complète du défect sans recourir à un matériel synthétique.

## Conclusion

La prise en charge des omphalocèles géantes n´est pas encore bien codifiée. Parmi les nombreux moyens proposés, le traitement conservateur à l´éosine aqueuse disodique 2% donne de bons résultats, des études à plus large échantillons devraient être encouragées compte tenu de l´accessibilité de l´éosine aqueuse dans les milieux à faibles revenus.

### Etat des connaissances sur le sujet

- Traitement conservateur à l´éosine aqueuse disodique est contre indiqué, une rupture du sac, une occlusion intestinale, une péritonite et une malformation associée;
- La cure radicale est le mode de traitement des omphalocèles de petit volume inférieur à 8 cm.

### Contribution de notre étude à la connaissance

- Nous avons apporté un schéma thérapeutique, des pansements humides à l´éosine aqueuse 2%, dilué dans sérum salé isotonique au début du traitement dans notre contrée;
- Une fermeture complète du défect sans recourir à un matériel synthétique dans les pays de faible revenu, en espérant au plus grand développement de la cavité abdominale.

## Remerciements

Remerciements aux membres du département de chirurgie des Cliniques Universitaires de Lubumbashi.
